# Dihydroartemisinin attenuates pulmonary inflammation and fibrosis in rats by suppressing JAK2/STAT3 signaling

**DOI:** 10.18632/aging.203874

**Published:** 2022-02-04

**Authors:** Xiaolan You, Xingyu Jiang, Chuanmeng Zhang, Kejia Jiang, Xiaojun Zhao, Ting Guo, Xiaowei Zhu, Jingjing Bao, Hongmei Dou

**Affiliations:** 1 Department of Gastrointestinal Surgery, Taizhou Clinical Medical School of Nanjing Medical University (Taizhou People’s Hospital), Taizhou 225300, Jiangsu, China; 2 Department of Oncology, The First Affiliated Hospital of Nanjing Medical University, Nanjing 210009, Jiangsu, China; 3 Department of Central Laboratory, Taizhou Clinical Medical School of Nanjing Medical University (Taizhou People’s Hospital), Taizhou 225300, Jiangsu, China; 4 Department of Respiratory Medicine, Taizhou Clinical Medical School of Nanjing Medical University (Taizhou People’s Hospital), Taizhou 225300, Jiangsu, China; 5 Department of the Pathology, Taizhou Clinical Medical School of Nanjing Medical University (Taizhou People’s Hospital), Taizhou 225300, Jiangsu, China; 6 Department of the Operation Room, Taizhou Clinical Medical School of Nanjing Medical University (Taizhou People’s Hospital), Taizhou 225300, Jiangsu, China

**Keywords:** pulmonary fibrosis, pulmonary inflammation, JAK2/STAT3 signaling, Coronavirus disease 2019, COVID-19

## Abstract

Coronavirus disease 2019 (COVID-19), caused by SARS-CoV-2, has induced a worldwide pandemic since early 2020. COVID-19 causes pulmonary inflammation, secondary pulmonary fibrosis (PF); however, there are still no effective treatments for PF. The present study aimed to explore the inhibitory effect of dihydroartemisinin (DHA) on pulmonary inflammation and PF, and its molecular mechanism. Morphological changes and collagen deposition were analyzed using hematoxylin-eosin staining, Masson staining, and the hydroxyproline content. DHA attenuated early alveolar inflammation and later PF in a bleomycin-induced rat PF model, and inhibited the expression of interleukin (IL)-1β, IL-6, tumor necrosis factor α (TNFα), and chemokine (C-C Motif) Ligand 3 (CCL3) in model rat serum. Further molecular analysis revealed that both pulmonary inflammation and PF were associated with increased transforming growth factor-β1 (TGF-β1), Janus activated kinase 2 (JAK2), and signal transducer and activator 3(STAT3) expression in the lung tissues of model rats. DHA reduced the inflammatory response and PF in the lungs by suppressing TGF-β1, JAK2, phosphorylated (p)-JAK2, STAT3, and p-STAT3. Thus, DHA exerts therapeutic effects against bleomycin-induced pulmonary inflammation and PF by inhibiting JAK2-STAT3 activation. DHA inhibits alveolar inflammation, and attenuates lung injury and fibrosis, possibly representing a therapeutic candidate to treat PF associated with COVID-19.

## INTRODUCTION

On 11 March 2020 World Health Organization (WHO) declared Coronavirus Disease 2019 (COVID-19), a respiratory disease caused by the SARS-CoV-2 virus, as a pandemic [1]. The latest figures from 12th December 2021 show that more than 270 million people have been infected with the virus, causing more than 5.32 million deaths worldwide. SARS-CoV-2 crosses borders and races, and infects patients of any age via person-to-person transmission [2]. The disease causes a wide range of symptoms, from asymptomatic to respiratory failure [3, 4]. Pulmonary fibrosis (PF) is a possible complication of pulmonary involvement in COVID-19, which is a chronic, progressive, and fatal form of fibrosing interstitial pneumonia [5, 6].

The pathophysiological characteristics of PF are the inability of damaged alveolar epithelial cells to reconstruct normally, excessive deposition of collagen (and other extracellular matrix components), and the persistent presence of fibroblasts, leading to the destruction of the normal lung structure [5]. The interstitial matrix widens during the progression of PF and the normal pulmonary parenchyma becomes compressed, leading to respiratory failure.

Radiological imaging is the fastest and most direct method to assess pulmonary parenchymal involvement [7–9]. Computed tomography (CT) is an important modality to evaluate PF. Some studies analyzed the chest CT characteristics of patients with COVID-19, and patchy ground-glass opacities were the most common changes [7]. The results of further imaging studies for COVID-19 further confirmed that PF might be one of the major complications in patients with COVID-19 [6, 10, 11].

PF comprises the formation of heterogeneous pulmonary lesions by the transformation of abnormal pulmonary epithelial cells and pulmonary interstitial cells into invasive pulmonary myofibroblasts, resulting in pulmonary parenchymal fibrosis. The specific mechanism of the origin and activation of invasive pulmonary myofibroblasts is still unknown. It is likely to be a multi-factor and multi-step process, including the recruitment of circulating fibroblasts’ blood mesenchymal precursors, activation of fibroblasts [12]. The process of PF involves the abnormal expression of multiple cytokines and the activation of many signaling pathways. Previous studies found that the Janus kinase 2 (JAK2)/signal transducer and activator of transcription 3 (STAT3) signaling pathways are involved in idiopathic pulmonary fibrosis (IPF) [13, 14]. The levels of activated JAK2 and STAT3 were elevated in alveolar type II epithelial cells (ATII) and lung fibroblasts from patients with IPF, in which JAK2 and STAT3 participate in lung fibrosis by dependent and independent mechanisms [12]. In addition, fibroblasts deficient in STAT3 were less sensitive to the pro-fibrotic effects of transforming growth factor beta 1 (TGF-β1) [15]. All of these studies suggested that STAT3 and its signaling pathways play an important role in PF.

STAT comprises an oncoprotein family encoded by tumor-associated genes and includes seven members. STAT3 can transduce peptide hormone signals from the cell surface to the nucleus and is activated by cytokines, growth factors, and many peptide hormones [16]. Upon binding of extracellular ligands to receptors on the surface of the cell membrane to form cytokine-receptor kinase complexes or growth factor-receptor complexes. These complexes can phosphorylate the intracellular kinases of the JAK or SRC families, then the activated JAK or SRC families can phosphorylate Tyr705 sites to activate the STAT3 [16].

Targeted inhibition of the STAT3 signaling pathway is expected to inhibit the formation of PF [16, 17]. For example, cryptotanshinone protects against PF through inhibiting STAT3 signaling pathway [18], AAV1.SERCA2a gene therapy reverses PF by blocking the STAT3 pathway [19]. Therefore, the development of drugs that can effectively block STAT3 phosphorylation and activation might represent one of the most promising strategies to treat PF. However, currently, there is no STAT3 inhibitor that can be used effectively and safely in the clinic. Dihydroartemisinin (DHA) is an artemisinin derivative that is used widely as an antimalarial. Accumulating evidence indicates that DHA can effectively inhibit STAT3 phosphorylation [20]. Experiments *in vitro* and *in vivo* confirmed that DHA is a potent STAT3 inhibitor, acting as an effective antimetastatic via inhibition of STAT3 phosphorylation and activation [20]. This study hypothesized that DHA could attenuate PF and thus we evaluated the effects of DHA in a rat bleomycin-induced PF model to determine its mechanism. The results of the present study could lead to a new treatment for patients with pneumonia and PF.

## RESULTS

### DHA attenuates early alveolar inflammation in a rat model of bleomycin-induced PF

**Figure 1:**
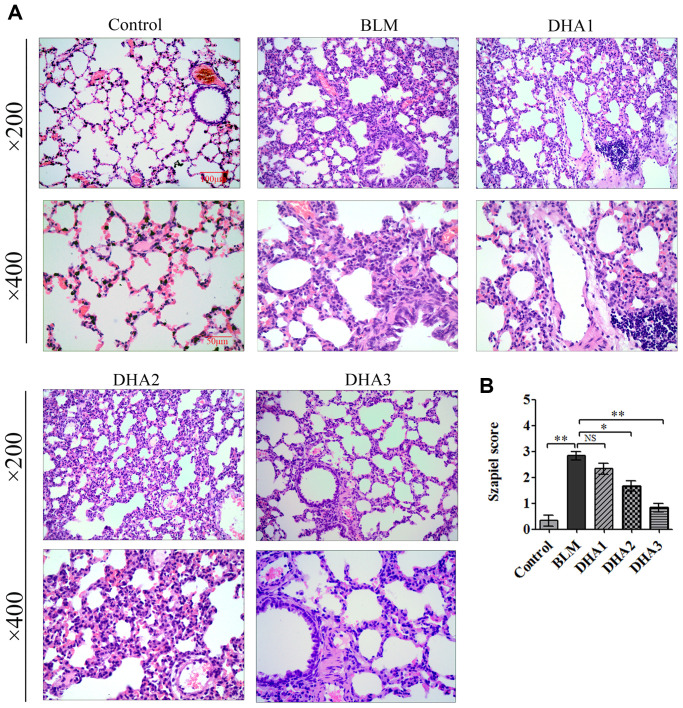
**DHA attenuates early alveolar inflammation in a bleomycin-induced rat pulmonary fibrosis model.** At 7 days after bleomycin intratracheal administration, with or without DHA treatment, the rats were sacrificed, and their lungs and blood were removed. (**A**) H&E staining was used to detect the lung histopathological changes and (**B**) Lung inflammation was scored (*n* = 6). ^*^P < 0.05, ^**^P < 0.001. Abbreviations: BLM: bleomycin; DHA-1: dihydroartemisinin 25 mg/kg/d; DHA-2: dihydroartemisinin 50 mg/kg/d; DHA-3: dihydroartemisinin 100 mg/kg/d; NS: not statistically significant.

At 7 days after intratracheal bleomycin (BLM) administration, with or without DHA treatment, the rats were sacrificed by inhaling excess isoflurane (*n* = 6/group). Lung tissue samples were removed quickly and blood samples were collected for further analysis. Alveolar inflammation was induced on day 7 after intratracheal administration of BLM. As visualized by hematoxylin and eosin (H&E) staining (Figure 1A), the lung tissues from the control group of rats had intact alveoli, while the lung tissues from the rats stimulated with BLM exhibited obvious inflammatory cell infiltration in the alveolar cavity, pulmonary edema accompanying septal thickness, damage to the lung architecture, and alveolar disarray. DHA attenuated alveolar inflammation and reduced the septal edema and alveolar damage. Lung tissues from the DHA-3 group (100 mg/kg/d) exhibited some infiltration of inflammatory cells and slight pulmonary edema in comparison to those in the BLM group.

The Szapiel scoring method was used to assess the extent of pulmonary inflammation; the scoring rules are shown in Table 1. Three sections per specimen were stained and evaluated for Szapiel scoring, and two independent pathologists, who were blinded to the experiment, assessed the results. Compared with that of the control group, the Szapiel scores of BLM group increased significantly. Compared with that of the BLM group, the Szapiel score of the DHA-2 (50 mg/kg/d) group and DHA-3 group decreased significantly (Figure 1B). In the early stage of the PF model, BLM caused pneumonia, and DHA effectively inhibited pulmonary inflammation. In addition, DHA inhibited pulmonary inflammation in a dose-dependent manner, with intraperitoneal injection of a high dose of DHA3 inhibiting alveolar inflammation significantly.

**Table 1:** Szapiel scoring rules for alveolar inflammation.

**Lever**	**Morphological analysis under the microscope**	**Scored**
1	No alveolar inflammation	0
2	Mild alveolitis, with an area less than 20% of the lung	1
3	Moderate alveolar inflammation, involving 20–50% of the lung	2
4	Severe alveolar inflammation, involving more than 50% of the lung	3

### DHA inhibits the inflammatory response in the lungs and serum of BLM-exposed rats

**Figure 2:**
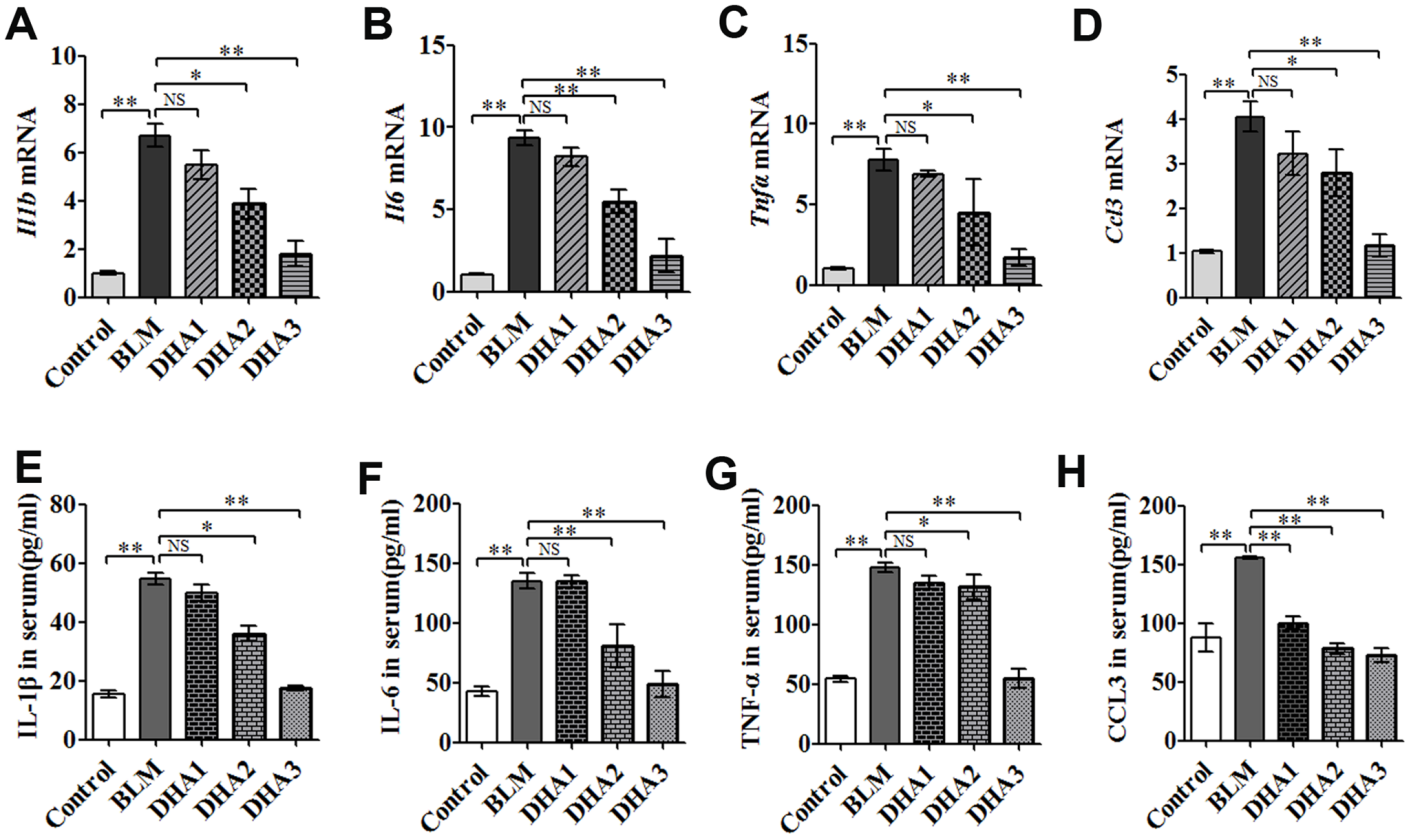
**DHA reduces the inflammatory response in the lungs and serum of bleomycin-exposed rats.** (**A**) *Il1b*, (**B**) *Il6*, (**C**) *Tnfa*, and (**D**) *Ccl3* mRNA levels in lung tissues were determined to assess the lung inflammatory response (*n* = 6), data were expressed as the fold change in mRNA expression normalized to *Gapdh* expression, with respect to the control group. (**E**) IL-1β, (**F**) IL-6, (**G**) TNFα, and (**H**) CCL3 protein levels in blood were determined to assess the lung inflammatory response (*n* = 6). ^*^P < 0.05, ^**^P < 0.001. Abbreviations: BLM: bleomycin; DHA-1: dihydroartemisinin 25 mg/kg/d; DHA-2: dihydroartemisinin 50 mg/kg/d; DHA-3: dihydroartemisinin 100 mg/kg/d; NS: not statistically significant; qRT-PCR: quantitative real-time reverse transcription PCR.

To reveal the role of DHA in the early inflammation of the BLM rat model, we used an enzyme-linked immunosorbent assay (ELISA) and quantitative real-time reverse transcription PCR (qRT-PCR) to detect inflammatory cytokines in the serum and lungs of rats at 7 days after modeling. The qRT-PCR result demonstrated that stimulation with BLM significantly increased the mRNA levels of interleukin (*Il*)*-1B*, *Il6*, tumor necrosis factor α (*Tnfa*), and chemokine (C-C Motif) Ligand 3 (*Ccl3*) in the rat lungs, while DHA attenuated these increases significantly (Figure 2A–2D) in a dose-dependent manner: A high dose of DHA3 inhibited the transcription of all the tested inflammatory cytokines in lung tissue effectively. Consistent with the qRT-PCR results, the protein levels of IL-1β, IL-6, TNFα, and CCL3 in the rat serum increased after BLM stimulation but decreased after DHA treatment (Figure 2E–2H). These results indicated that DHA attenuated the inflammatory response in the lungs and serum of BLM-exposed rats.

### DHA reduces the inflammatory response in the lungs via JAK2/STAT3 signaling

**Figure 3:**
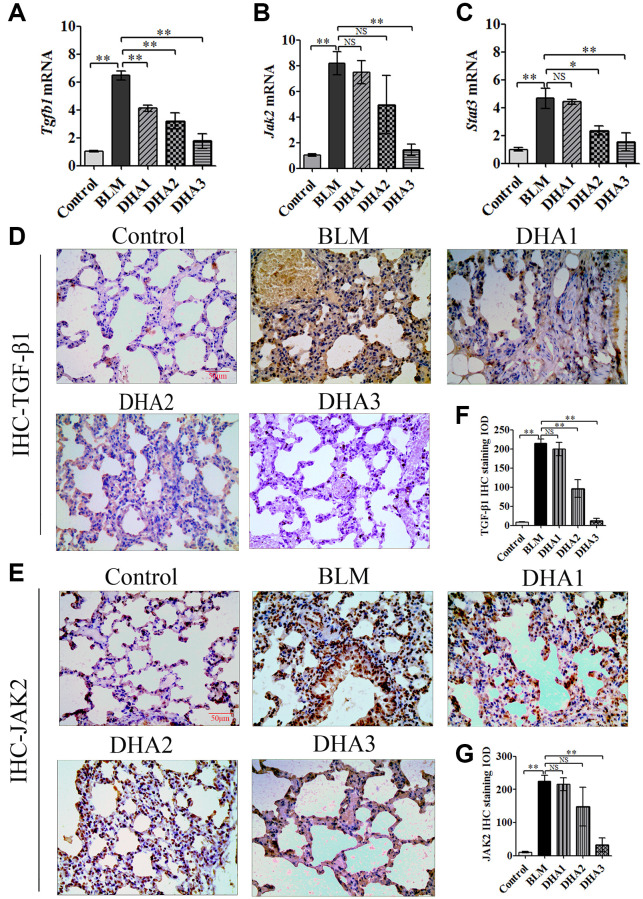
**DHA inhibited the JAK2/STAT3 pathway in pneumonia tissue.** (**A**) *Tgfb1*, (**B**) *Jak2*, and (**C**) *Stat3* mRNA levels in the lungs were determined using qRT-PCR (*n* = 6), data are expressed as the fold change in mRNA expression normalized to *Gapdh* expression, with respect to the control group. Representative images of (**D**) TGF-β1 and (**E**) JAK2 protein expression in lung tissue detected using IHC. Mean IOD of (**F**) TGFβ1 and (**G**) JAK2 protein by IHC staining. Data are expressed as the means ± standard error. ^*^P < 0.05, ^**^P < 0.001. Abbreviations: BLM: bleomycin; DHA-1: dihydroartemisinin 25 mg/kg/d; DHA-2: dihydroartemisinin 50 mg/kg/d; DHA-3: dihydroartemisinin 100 mg/kg/d; IHC: immunohistochemistry; IOD: integrated optical density; NS: not statistically significant.

**Figure 4:**
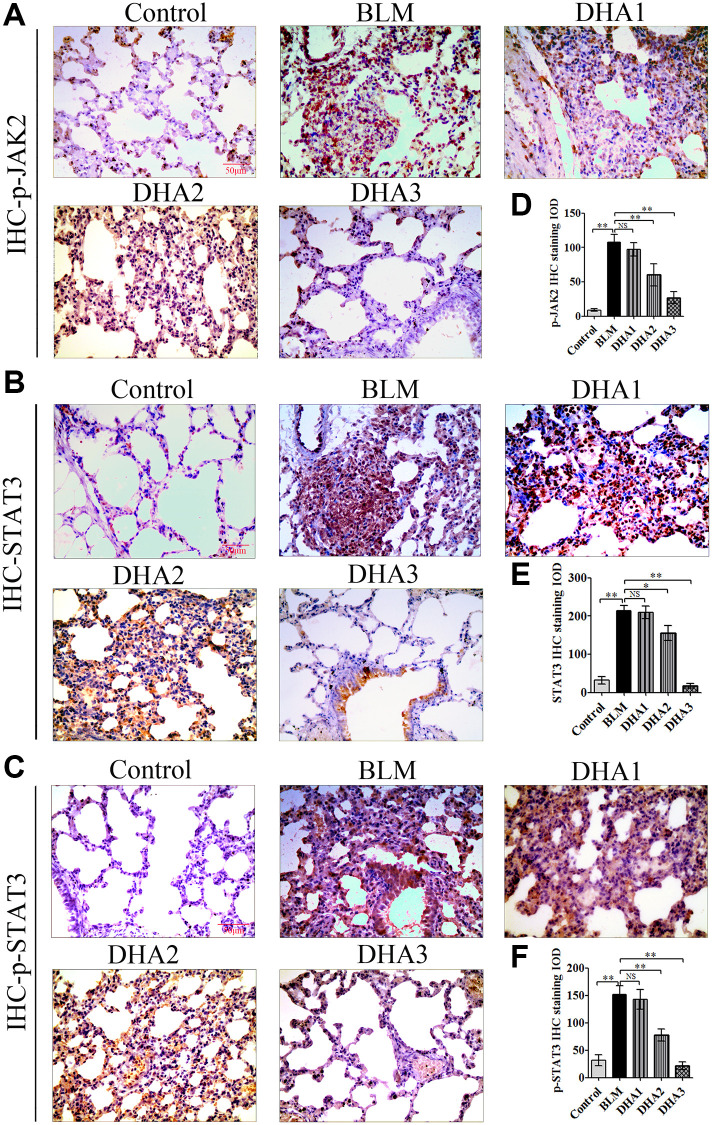
Representative images of (**A**) p-JAK2, (**B**) STAT3, and (**C**) p-STAT3 protein levels in lung tissue detected by IHC. Mean IOD of (**D**) p-JAK2, (**E**) STAT3, and (**F**) p-STAT3 protein by IHC staining. Data are expressed as the means ± standard error. ^*^P < 0.05, ^**^P < 0.001. Abbreviations: BLM: bleomycin; DHA-1: dihydroartemisinin 25 mg/kg/d; DHA-2: dihydroartemisinin 50 mg/kg/d; DHA-3: dihydroartemisinin 100 mg/kg/d; IHC: immunohistochemistry; IOD: integrated optical density; NS: not statistically significant.

To explore the mechanism by which DHA inhibits the early alveolar inflammatory response in the rat BLM-induced PF model, we evaluated the *Tgfb1*, *Jak2*, and *Stat3* mRNA expression and TGF-β1, JAK2, p-JAK2, STAT3, and p-STAT3 protein levels in lung tissues at day 7 after intratracheal administration of BLM, with and without DHA treatment, using qRT-PCR and immunohistochemistry staining. In homogenized lung tissue, the *Tgfb1*, *Jak2*, and *Stat3* mRNA levels in the BLM group were higher than those in the control groups. DHA1 (25 mg/kg/d) inhibited the expression of *Tgfb1* mRNA significantly (Figure 3A), DHA3 inhibited the expression of *Jak2* mRNA significantly (Figure 3B), and DHA2 inhibited the expression of *Stat3* mRNA significantly (Figure 3C). The IHC results also showed that BLM increased the levels of TGF-β1, JAK2, p-JAK2, STAT3, and p-STAT3 in lung tissues (Figure 3D–3E, Figure 4A–4C), and the mean integrated optical density (IOD) values showed that DHA reduced the levels of TGF-β1, JAK2, p-JAK2, STAT3, and p-STAT3 proteins in a dose-dependent manner (Figure 3F–3G, Figure 4D–4F). DHA could inhibit the phosphorylation of JAK2, thereby inhibiting the phosphorylation of STAT3. Thus, DHA might reduce the inflammatory response of the lungs by inhibiting the activation of JAK2/STAT3 signaling.

### DHA reduces PF induced by BLM in rats

**Figure 5:**
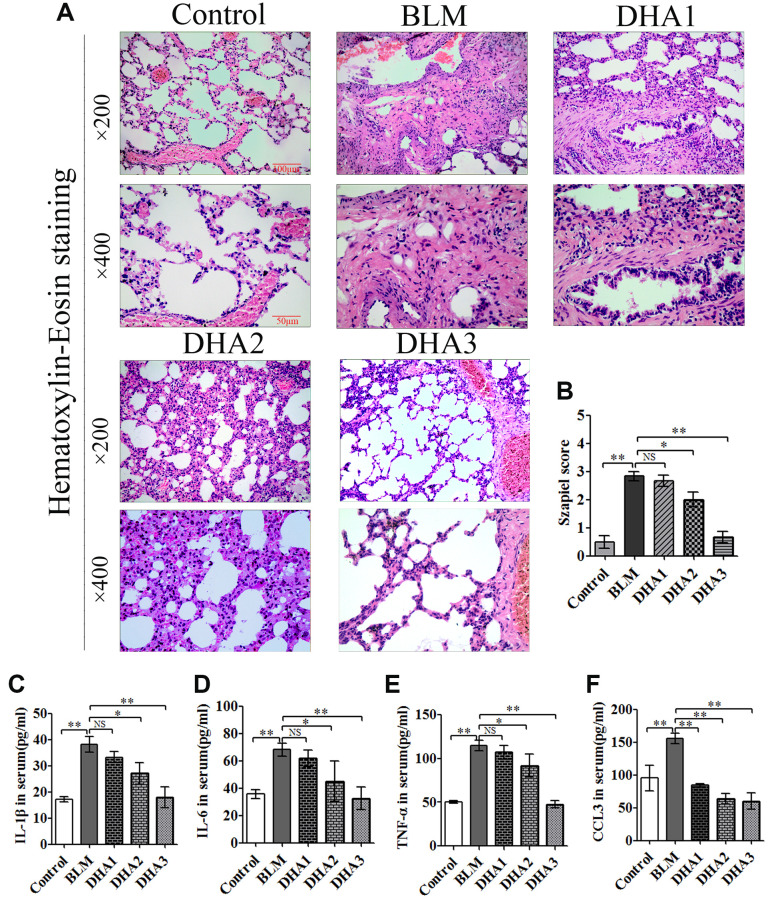
**DHA reduces bleomycin-induced pulmonary fibrosis in rats.** Rats were sacrificed at 28 days after the injection of bleomycin, with or without DHA treatment. Their blood and lungs were removed. (**A**) H&E staining of bleomycin-treated rat lungs, with and without DHA treatment. (**B**) Lung inflammation was scored (*n* = 6). (**C**) IL-1β, (**D**) IL-6, (**E**) TNFα, and (**F**) CCL3 protein in serum were determined using ELISA (*n* = 6). Data are expressed as the means ± standard error. ^*^P < 0.05, ^**^P < 0.001. Abbreviations: BLM: bleomycin; DHA-1: dihydroartemisinin 25 mg/kg/d; DHA-2: dihydroartemisinin 50 mg/kg/d; DHA-3: dihydroartemisinin 100 mg/kg/d; NS: not statistically significant; H&E: hematoxylin and eosin; ELISA: enzyme-linked immunosorbent assay.

On day 28 after intratracheal administration of BLM, other rats were sacrificed by inhaling excess isoflurane (*n* = 6/group). H&E staining was performed to explore the pathological alterations and Masson staining was used to evaluate the collagen fibrils in the lung tissues., Compared with those of the control group, under the microscope, the lung tissues in the BLM group showed loss of alveolar architecture, pulmonary septal thickening, and increased cell number, ultimately resulting in severe lung tissue damage. However, DHA attenuated the pathological alterations in the lung tissues at a dose of DHA2 (Figure 5A). The Szapiel score also showed that DHA inhibited alveolar inflammation, with the Szapiel score decreasing in a DHA dose-dependent manner (Figure 5B). ELISA was used to explore the inflammatory cytokines in the serum of all rats, which demonstrated significantly higher cytokine levels in BLM group than those in the control group. By contrast, the IL-1β, IL-6, TNFα, and CCL3 levels in serum were reduced by DHA in a dose dependent manner, above 50 mg/kg/d suppressed all the tested inflammatory cytokines levels, and all doses of DHA reduced CCL3 levels (Figure 5C–5F).

**Figure 6:**
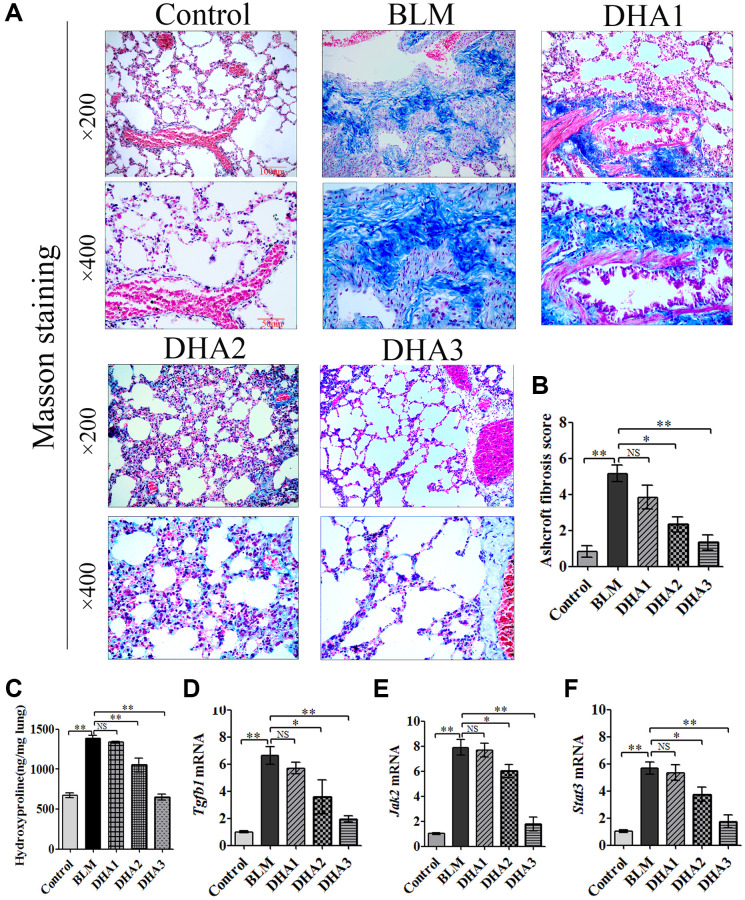
**DHA reduced collagen deposition in pulmonary fibrosis tissues and inhibited the expression of *Tgfb1*, *Jak2*, and *Stat3* mRNA in lung tissues.** The rats were sacrificed at 28 days after bleomycin injection, with or without DHA treatment, their lungs were removed and blood collected. (**A**) Representative images of Masson staining of bleomycin-treated rat lungs, with and without DHA treatment. (**B**) Evaluation of pulmonary fibrosis using the Ashcroft score (*n* = 6). (**C**) Determination of hydroxyproline in lung tissue (*n* = 6). (**D**) *Tgfb1*, (**E**) *Jak2*, and (**F**) *Stat3* mRNA levels in lung tissues were determined using qRT-PCR (*n* = 6), data are expressed as fold change in mRNA expression normalized to *Gapdh* expression, with respect to the control group. ^*^P < 0.05, ^**^P < 0.001. Abbreviations: BLM: bleomycin; DHA-1: dihydroartemisinin 25 mg/kg/d; DHA-2: dihydroartemisinin 50 mg/kg/d; DHA-3: dihydroartemisinin 100 mg/kg/d; NS: not statistically significant; qRT-PCR: quantitative real-time reverse transcription PCR.

Masson staining showed massive deposits of collagen in the pulmonary septum (blue) of lung tissues in the BLM group, and DHA treatment reduced the size of the collagen deposits (Figure 6A). The Ashcroft score was used to evaluate the pathological alterations of PF (Table 2). The Ashcroft score of the BLM group was increased significantly, and DHA decreased the Ashcroft score in a dose-dependent manner Figure 6B). The hydroxyproline content in the lung tissue was measured to evaluate PF. At 28 days after BLM treatment, the rats demonstrated a higher hydroxyproline content than that in the control group. Compared with that of the BLM group, DHA reduced the hydroxyproline content significantly in a dose-dependent manner (Figure 6C).

**Table 2:** Ashcroft scoring rules for PF.

**Lever**	**Morphological analysis under the microscope**	**Scored**
0	Normal	0
1	Partially enlarged alveoli, alveolar septum thickened slightly (≤3 times the normal)	1
2	Alveolar septum thickened moderately (>3 times the normal), without damage to the lung architecture	2
3	Alveolar septum thickened moderately (>3 times the normal), and increased fibrotic tissue increased	3
4	The area of fibrous tissue mass is less than 10% of the lung, with mild lung structural damage	4
5	The area of fibrous tissue mass is 10–50% of the lung, and with pulmonary structural damage	5
6	The area of fibrous tissue mass is more than 50% of the lung, and with obvious pulmonary structural damage	6
7	Severe lung damage, large areas of fibrosis, honeycomb lung	7
8	Full field fibrous tissue	8

### DHA inhibits PF via JAK2/STAT3 signaling

TGF-β1 is known to be critical for PF; therefore, we used qRT-PCR to detect *Tgfb1* mRNA expression in the lung tissue of all rat groups. The results confirmed that the expression of *Tgfb1* mRNA was higher than that in the control group on day 28 after BLM injection. However, DHA2 could significantly reduce this increase (Figure 6D). We also detected the mRNA of *Jak2* and *Stat3* in the rat lung tissues. Similar to the results for *Tgfb1*, high dose DHA2 could decrease the expression of *Jak2* and *Stat3* mRNA significantly (Figure 6E and 6F).

**Figure 7:**
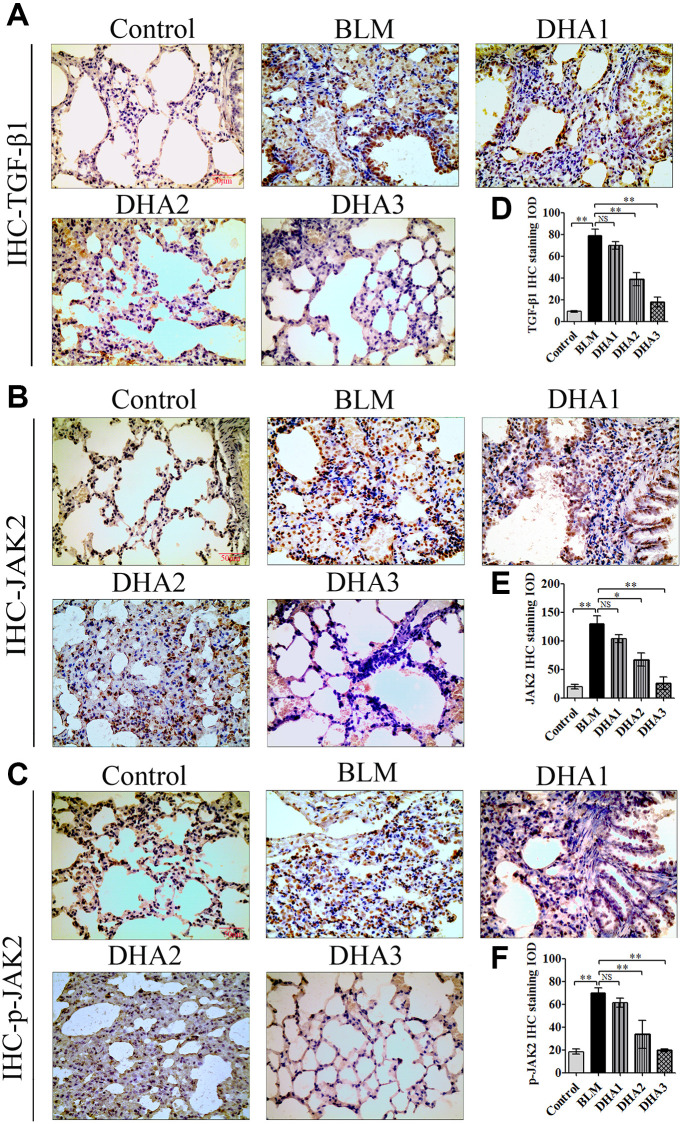
DHA inhibited pulmonary fibrosis via the JAK2/STAT3 pathway (**A**) Representative images of (**A**) TGF-β1, (**B**) JAK2, and (**C**) p-JAK2 proteins in lung tissues, detected using IHC. Mean IOD of (**D**) TGF-β1, (**E**) JAK2, and (**F**) p-JAK2 IHC staining. Data are expressed as the means ± standard error. ^*^P < 0.05, ^**^P < 0.001. Abbreviations: BLM: bleomycin; DHA-1: dihydroartemisinin 25 mg/kg/d; DHA-2: dihydroartemisinin 50 mg/kg/d; DHA-3: dihydroartemisinin 100 mg/kg/d; IHC: immunohistochemistry; IOD: integrated optical density; NS: not statistically significant.

**Figure 8:**
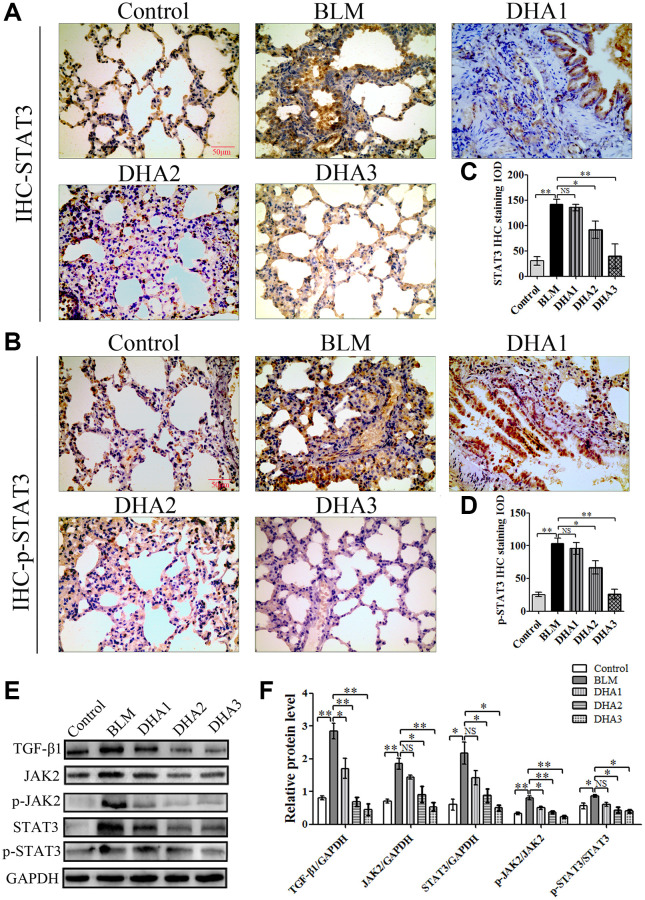
(**A**) STAT3, and (**B**) p-STAT3 protein in lung tissues, detected using IHC. Mean IOD of (**C**) STAT3, and (**D**) p-STAT3 IHC staining. Data are expressed as the means ± standard error. (**E**) and (**F**) Western blotting analysis of TGF-β1, JAK2, p-JAK2, STAT3, and p-STAT3 proteins in lung tissues. (All *n* = 6). ^*^P < 0.05, ^**^P < 0.001. Abbreviations: BLM: bleomycin; DHA-1: dihydroartemisinin 25 mg/kg/d; DHA-2: dihydroartemisinin 50 mg/kg/d; DHA-3: dihydroartemisinin 100 mg/kg/d; IHC: immunohistochemistry; IOD: integrated optical density; NS: not statistically significant.

IHC was used to detect the levels of TGF-β1, JAK2, p-JAK2, STAT3, and p-STAT3 proteins in lung tissues (Figure 7A–7C, Figure 8A and 8B). According to the mean IOD score, DHA2 reduced the levels of TGF-β1, JAK2, p-JAK2, STAT3, and p-STAT3 significantly (Figure 7D–7F, Figure 8C and 8D).

To further confirm that DHA inhibited PF through JAK2/STAT3 signaling, the levels of TGF-β1, JAK2, p-JAK2, STAT3, and p-STAT3 were measured using western blotting in lung tissues. We found that DHA could reduce the levels of TGF-β1, JAK2, STAT3, p-JAK2, and p-STAT3 significantly (Figure 8E and 8F).

## DISCUSSION

PF is a common pathological process after lung tissue injury, and chronic relapses of PF affects the quality of life for patients, even causing respiratory failure. PF occurs after severe pulmonary infection caused by SARS-CoV-2 [21]. Currently, COVID-19 is prevalent worldwide; however, there is still no effective drug treatment for PF. Therefore, exploring the mechanism of PF to find effective drugs is expected to improve the quality of life for patients with COVID-19.

Pulmonary inflammation is the initial cause of PF [21]. During the inflammatory response, the alveolar epithelium and vascular endothelium are damaged initially, and inflammatory cells and immune effector cells enter the lung to release inflammatory mediators, resulting in the formation of alveolar exudates, including a variety of inflammatory cytokines [22]. In the present study, in the early stage of rat BLM-induced PF model, the mRNA and protein levels of inflammatory factors IL-1β, IL-6, TNFα, and CCL3 in the lung tissues and serum were increased significantly.

CCL3 is expressed on the surface of macrophages and can directly interfere with the secretion of TGF-β1, thus regulating the course of acute pulmonary inflammation [23]. Normally, the expression of CCL3 is low; however, when the target cells are stimulated by endotoxins, such as lipopolysaccharide, viral proteins, or proinflammatory factors (IL-1β), the related cell signaling pathway is activated, and CCL3 expression increases. The earliest reports of COVID-19 clarified that IL-1β, IL-6, TNFα, and CCL3 in the peripheral blood of patients with severe COVID-19 pneumonia were significantly higher than those of non-severe patients, and this cytokine storm is an important factor contributing to death from COVID-19 [24]. In addition, our study found that IL-1β, IL-6, TNFα, and CCL3 levels in rat blood increased significantly during the acute inflammation stage of PF induced by BLM. However, DHA could reduce the inflammatory response in the lungs of the model rats significantly and reduced the levels of cytokines, such as CCL3, in the blood.

Previous studies have shown that CCL3 and its receptors promote fibrosis [23], and analysis of alveolar lavage fluid in patients with congenital PF showed an increased level of CCL3. Our study also found that the CCL3 level in the blood of rats with PF increased significantly. However, the CCL3 level in the blood of rats treated with DHA2 decreased significantly. These findings suggested that CCL3 plays an important role in pulmonary inflammation and PF, and that DHA inhibits CCL3 expression to attenuate pulmonary inflammatory responses and PF.

Cytokines can either directly activate myofibroblasts or induce the transformation of epithelial cells into fibroblasts, leading to the formation of fibrosis [25]. Furthermore, many cytokines, particularly the IL-6 family, can activate STAT3 rapidly within cells. Persistent STAT3 activation results in chronic inflammation and fibrosis [16].

In the early stage of BLM-induced PF, we observed the activation of the JAK2/STAT3 signaling pathway [26]. JAK/STAT signaling induces macrophage activation, and the activated macrophages secrete cytokines, such as platelet-derived factor, TNFα, IL-28, and nitric oxide. TNFα induces the expression of TGF-β directly [27]; therefore, in the early stage of the BLM-induced rat model, the expression of TGF-β1 was also high in lung tissue. TGF-β1 is a key factor for fibrosis that can promote the transformation of pulmonary fibroblasts into myofibroblasts, produce more matrix protein components, and release them into the extracellular matrix, leading to PF [28, 29]. Furthermore, TGF-β1 signaling can induce the phosphorylation and activation of JAK2, thereby activating the JAK2/STAT3 signaling pathway, and this vicious circle amplifies the inflammatory response and PF [26]. Our results showed that in the later stage of the PF model induced by BLM, Masson staining confirmed the presence of PF, and IHC staining verified the activation of JAK2/STAT3 signaling. Our findings suggested that JAK2/STAT3 signaling pathways play a crucial role in the early stage of the inflammatory response in the BLM-induced PF model, as well as in the later stage of PF. Previous studies have detected JAK2/p-JAK2 and STAT3/pSTAT3 levels using quantitative real time-PCR, western blotting, and immunohistochemistry, and the levels of these proteins were observed to be upregulated in the lung tissues of patients with IPF, which demonstrated that phosphorylated STAT3 participates in both lung epithelial cell damage and the fibroblast to myofibroblast transition, making STAT3 an attractive therapeutic target in PF [12, 26]. Some researchers have attempted to interfere with STAT3 activation and associated signaling as a strategy to ameliorate PF, and they found that a cell-permeable peptide inhibitor of STAT3 phosphorylation or STAT3 associated signaling pathway inhibitors might be a feasible therapeutic option for PF [30, 31].

A growing number of STAT3 inhibitors have been developed and tested. Artemisinin is an active component extracted from the leaves and buds of *Artemisia annua*, a traditional Chinese medicine plant, and is a sesquiterpene lactone compound containing internal peroxides. Used in the treatment of various types of malaria, with high efficiency, Artemisinin (or its derivative DHA) induces few side effects and has other beneficial characteristics [32]. DHA has many pharmacological effects on viral infection [33], inflammation [34], and tumor proliferation [35]. In addition, DHA also shows a relatively good safety profile.

Previous studies have found that DHA can effectively block the phosphorylation of STAT3, thereby inhibiting its activation, with negligible toxicities [36]. In addition, DHA has been reported to exert protective effects against lung inflammation [37, 38]. The present study revealed the role of DHA in the early inflammatory response in a rat model of BLM-induced PF. DHA effectively alleviated lung tissue damage and inhibited the release of inflammatory mediators. Notably, DHA attenuated pulmonary inflammatory responses in a dose-dependent manner, such that lung tissues from the rats treated with 100 mg/kg/d of DHA exhibited only slight infiltration of inflammatory cells and little pulmonary edema, suggesting a therapeutic effect on BLM-induced acute lung injury. Our findings suggested that DHA is a potential therapeutic agent for patients with PF. Further experiments demonstrated that DHA reduced lung inflammation and lung tissue damage by inhibiting the phosphorylation of JAK2 and STAT3. The levels of phosphorylated JAK2 and phosphorylated STAT3 were reduced significantly in lung tissue by daily injection of 100 mg/kg DHA. In the late stage of the BLM-induced PF, we also found that DHA inhibited collagen deposition significantly in the lung tissues of the rat model, as well as the content of hydroxyproline, indicating that DHA inhibited the formation of PF. Further experiments showed that DHA inhibited TGF-β1 expression and the phosphorylation of JAK2 and STAT3 significantly.

The effect of DHA on JAK2 has been studied [39]; however, the present study further revealed that DHA inhibited STAT3 phosphorylation by inhibiting JAK2 phosphorylation. Regarding the mechanism by which DHA inhibits JAK2 phosphorylation, our study found that TGF-β1 expression and TNFα expression decreased significantly in lung tissues after treatment with DHA.

## CONCLUSIONS

We concluded that DHA might reduce the expression of TNF-α, IL-6, and TGF-β1, and inhibit the inflammatory response and PF through the TGF-β/JAK2/STAT3 signaling pathway. The COVID-19 pandemic is causing severe health, economic, and social challenges to the global population. Therefore, it is important to anticipate and prepare for this challenge. PF is an adverse outcome of many viral infections, leading to acute respiratory distress syndrome. Currently, there is a lack of effective and safe drugs with antiviral, anti-inflammatory, and anti-fibrosis properties. DHA has the potential to treat COVID-19-associated PF and prevent the long-term consequences of fibrosis that the epidemic may cause. Finally, we hope that our findings will lead to the development of drugs that can prevent or treat COVID-19 induced PF.

## MATERIALS AND METHODS

### Ethics statement

The study was conducted according to the guidelines of the Declaration of Helsinki, and approved by the Ethics Committee of Yang Zhou University (YZU-EC-202112004, Jiangsu, China). The experiments were performed in accordance with the requirements of the ARRIVE (Animal Research: Reporting *In Vivo* Experiments) guideline. Rats were anesthetized with isoflurane, and all necessary efforts were taken to minimize suffering prior to the experiments.

### Animal experiments

Five-week-old male Wistar rats weighing 110–130 g were used in all experiments. The rats were bought from the Comparative Medicine Centre of Yangzhou University (Yang Zhou, Jiangsu, China). The rats were bred under pathogen-free conditions with a 12-hour dark/light cycle, in a laminar flow cabinet at 24°C and a relative humidity of 40–60%. Rats were provided with food and purified water, and were allowed 1 week to adapt to the environmental before the experiment.

The rats were randomly divided into five groups (*n* = 12 in each group) as follows: 1. The BLM group received bleomycin (BLM; 5 mg/kg) via intratracheal instillation, BLM was dissolved in 0.9% NaCl at a concentration of 4 mg/ml; 2. The Control group, who received the same volume of 0.9% NaCl solution via intratracheal installation only; 3. DHA groups received intratracheal instillation of BLM, then received daily intraperitoneal injection of DHA from second day. The DHA1 group received daily intraperitoneal injection of DHA1 (25 mg/kg/d), DHA was dissolved in dimethyl sulfoxide (DMSO) at a concentration of 30 mg/ml; 4. The DHA2 group, who received intratracheal instillation of BLM and daily intraperitoneal injection of DHA2 (50 mg/kg/d), DHA was dissolved in DMSO at a concentration of 60 mg/ml; and 5. The DHA3 group, who received intratracheal instillation of BLM and daily intraperitoneal injection of DHA3 (100 mg/kg/d), DHA was dissolved in DMSO at a concentration of 120 mg/ml. To exclude the intervention effect of DMSO on pneumonia and PF, we adjusted the concentration of DHA to ensure equal doses of DMSO in each group. BLM was injected directly into the trachea under direct vision after the trachea had been exposed through a midline anterior neck incision.

### Hydroxyproline and collagen measurement

The content of hydroxyproline was measured to calculate the total collagen content in lung tissue. Hydroxyproline determination kit (A030-2-1) was purchased from Jiancheng Bioengineering Institute (Nanjing, Jiangsu, China), which was operated in strict compliance with the manufacturer's instructions. All experiments were performed in triplicate. Data for the hydroxyproline content are expressed as nanograms of hydroxyproline per milligrams of lung tissue (ng/mg lung).

### Cytokine measurements

The levels of IL-1β, IL-6, TNFα, and CCL3 in rat sera were detected using ELISA kits. The rat IL-1β enzyme-linked immunosorbent assay (ELISA) Kit (70-EK301B/3-96), the rat IL-6 ELISA Kit (70-EK306HS-96), the rat TNFα ELISA Kit (70-EK382/3-96), and the rat CCL3 ELISA Kit (70-EK361-96) were purchased from Lianke Bio (Hangzhou, Zhejiang, China). All experiments were operated according to the manufacturer’s instructions and the assays were performed in triplicate.

### RNA extraction and quantitative real-time reverse transcription PCR (qRT-PCR)

Total RNA was extracted from lung tissue using a RNeasy Mini Kit (Invitrogen, Waltham, MA, USA). A reverse transcription kit (Takara, Shiga, Japan) was used to perform first-strand cDNA synthesis, and an iQ5 Multicolor Real-Time PCR Detection System (Bio-Rad, Hercules, CA, USA) was used to conduct the quantitative real-time PCR, using SYBR Green dye (Roche Diagnostics, Mannheim, Germany). The *Gapdh* (glyceraldehyde-3-phosphate dehydrogenase) gene was used as an internal control to calculate the relative fold expression levels. The quantitative real-time PCR was performed in triplicate. Primers for *Il1B* (IL-1β), *Il6*, *Tnfa* (TNFα), *Ccl3*, *Tgfb1* (TGF-β1), *Jak2*, *Stat3*, and *Gapdh* are shown in Table 3.

**Table 3:** Primers used for qRT-PCR in the present study.

**Gene**	**Primer sequence (5′ to 3′)**
*Il1b*	F: AATCTCACAGCAGCATCTCGACAAG
	R: TCCACGGGCAAGACATAGGTAGC
*Il6*	F: ACTTCCAGCCAGTTGCCTTCTTG
	R: TGGTCTGTTGTGGGTGGTATCCTC
*Tnfa*	F: AAAGGACACCATGAGCACGGAAAG
	R: CGCCACGAGCAGGAATGAGAAG
*Ccl3*	F: CTGAGATTAGAGGCAGCAAGGAACC
	R: TGAAGAGTCCCTGGATGTGGCTAC
*Tgfb1*	F: GACCGCAACAACGCAATCTATGAC
	R: CTGGCACTGCTTCCCGAATGTC
*Jak2*	F: GTGTGGAGATGTGCCGCTATGAC
	R: GGAGATGCTCTTCCGTGCTGTG
*Stat3*	F: GAACTGAGTGAGCGTGGGTGATG
	R: AGGACAGGCGGACAGAACATAGG
*Gapdh*	F: GGCACAGTCAAGGCTGAGAATG
	R: ATGGTGGTGAAGACGCCAGTA

### Immunohistochemistry (IHC)

IHC was performed using lung tissues. Anti-phospho-JAK2 (Y1007) antibodies were purchased from Abcam (ab195055, Cambridge, UK); anti-JAK2 and anti-phospho-STAT3 (Tyr705) antibodies were purchased from Cell Signaling Technology (3230S and 9145S, Danvers, MA, USA); anti-STAT3 (bsm-33218M) and anti-TGF-β1 (bs0086R) antibodies were purchased from Bioss (Beijing, China). Primary antibodies recognizing TGF-β1 (dilution, 1:200), JAK2 (dilution, 1:200), p-JAK2 (dilution, 1:100), STAT3 (dilution, 1:200), or p-STAT3 (dilution, 1:200) were incubated with tissue slides overnight at 4°C, and then processed using standard procedures. All staining was imaged digitally using the same light exposure and evaluated using Image Pro Plus, a digitalized IHC scoring program (Media Cybernetics, San Diego, CA, USA). All experiments were performed in triplicate. The immunostaining results are expressed as the mean IOD.

### Histological analysis

H&E and Masson staining were used to analyze the grades of pulmonary inflammation and fibrosis. Lung tissues were fixed using 4% paraformaldehyde for 24 h at room temperature and then embedded in paraffin. Sections for pathological analysis were cut at 4 μm thickness and stained with H&E, Masson staining, and using immunohistochemistry. Masson’s trichrome was purchased from Beijing Solarbio Science and Technology, Ltd., (GB1340, Solarbio, Beijing, China). An optical microscope was used to observe the H&E and Masson stained sections and lung injury was scored by two pathologists who were blinded to the treatment. The severity of pulmonary inflammation was scored using the Szapiel scoring method [40] and PF was scored using the Ashcroft scoring system [41]; (Tables 1 and 2).

### Western blotting

The proteins extracted from tissues were separated using sodium dodecyl sulfate-polyacrylamide gel electrophoresis and the separated proteins were transferred onto a nitrocellulose membrane (GE Healthcare Life Sciences, Pittsburgh, PA, USA). Blots were probed with anti-TGF-β1, anti-JAK2, anti-p-JAK2, anti-STAT3, anti-p-STAT3, or anti-GAPDH primary antibodies at a dilution of 1:2,000. The anti-GAPDH antibody (bs-0755R) was provided by Bioss (Beijing, China). The secondary HRP-conjugated antibody was also used at a dilution of 1:2,000. The West Pico chemiluminescent Substrate (Pierce, Carlsbad, CA, USA) was used to visualize the protein bands and densitometric image analysis software (Image Master VDS; Pharmacia Biotech) was used to quantify the protein bands. All assays were performed in triplicate.

### Statistical analysis

SPSS 20.0 (IBM Corp., Armonk, NY, USA) was used to conduct the statistical analysis. The means ± standard error was used to express the continuous variables, one-way analysis of variance (ANOVA) and Dunnett’s *t* test was used for comparisons between groups based on the normal distribution of the data. Values of *P* < 0.05 were regarded as statistically significant (^*^P < 0.05, ^**^P < 0.001).
